# Supplementary material for: I Like This New Me: Unravelling Population Structure of Mediterranean Electric Rays and Taxonomic Uncertainties within Torpediniformes
Source: Animals (Basel). 2023 Sep 13;13(18):2899. doi: 10.3390/ani13182899 (PMC10525375; doi:10.3390/ani13182899)
Supplement: Supplementary file 1 [file animals-13-02899-s001.zip › Figure S1_BAPS.pdf]

Figure S1. BAPS

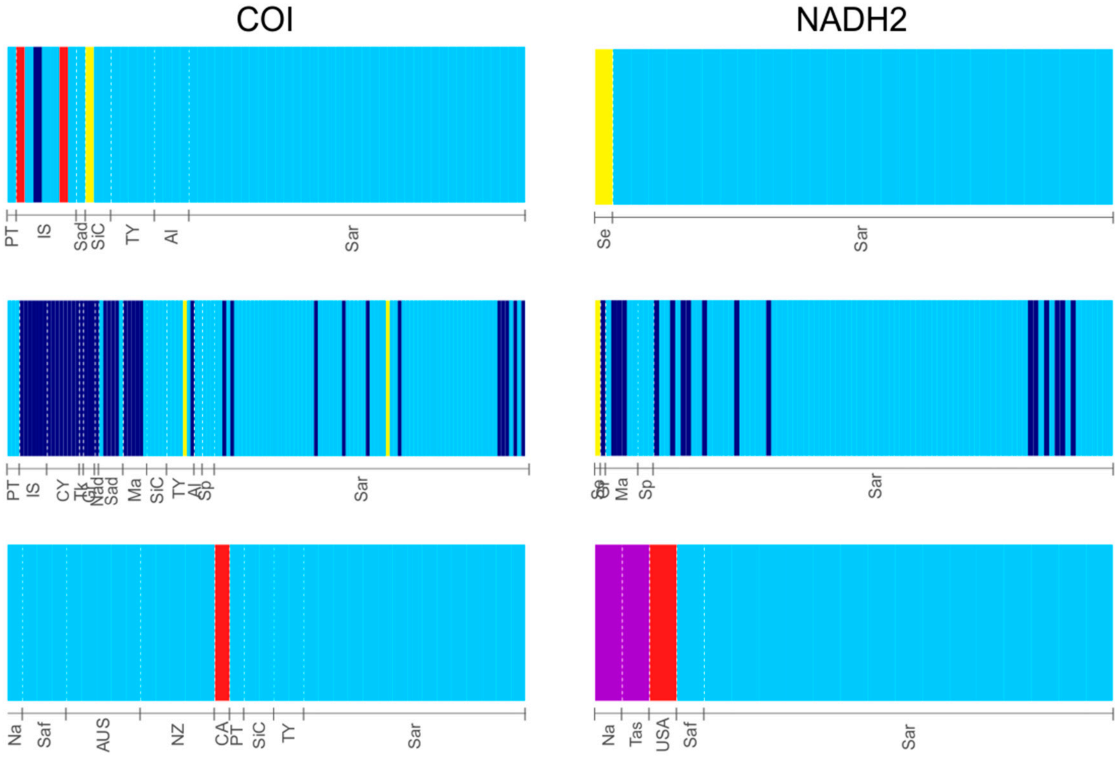

Figure S1. COI and NADH2 outputs performed using BAPS for *T. torpedo* (top), *T. marmorata* (middle) and *T. nobiliana* (bottom). The codes for the locations are described in the caption of SM4. Sar= Sardinian samples.
