# Supplementary material for: I Like This New Me: Unravelling Population Structure of Mediterranean Electric Rays and Taxonomic Uncertainties within Torpediniformes
Source: Animals (Basel). 2023 Sep 13;13(18):2899. doi: 10.3390/ani13182899 (PMC10525375; doi:10.3390/ani13182899)
Supplement: Supplementary file 1 [file animals-13-02899-s001.zip › Tables S4_AMOVA.pdf]

SM 4. AMOVA

Table S4.1. Results of AMOVA for COI, and NADH2 datasets for the *T. marmorata* Sardinian sequences. *** = P-values < 0.001; ** = P-value <0.01; * = P-value <0.05. Between parentheses are the locations as shown in Figure 1. S= south, SW = southwestern, N = north, NW = northwestern, E = east, NE = northeast, W = west.

T. marmorata COI (Sardinia)			
Source of variation	Percentage of variation	Fixation Index	P-value
**One group**			
Among populations	14.90	Φ_{ST} = 0.149	**
Within populations	85.10		
**Two groups (N+SW+NW+W / S+E+NE)**			
Among groups	-15.66	Φ_{CT} = -0.15658	ns
Among populations	26.83	Φ_{SC} = 0.23196	**
Within populations	88.83	Φ_{ST} = 0.11170	**
**Three groups (N+NE+NW / W+SW/S+E)**			
Among groups	-0.71	Φ_{CT} = 0.007	ns
Among populations	20.39	Φ_{SC} = 0.202	***
Within populations	80.32	Φ_{ST} = 0.197	***

T. marmorata NADH2 (Sardinia)			
**One group**			
Among populations	13.08	Φ_{ST} = 0.131	**
Within populations	86.92		
**Two groups (N+SW+NW+W / S+E+NE)**			
Among groups	-8.98	Φ_{CT} = -0.089	ns
Among populations	19.79	Φ_{SC} = 0.181	**
Within populations	89.19	Φ_{ST} = 0.108	**
**Three groups (N+NE+NW / W+SW/S+E)**			
Among groups	0.33	Φ_{CT} = 0.003	ns
Among populations	4.38	Φ_{SC} = 0.047	ns
Within populations	95.3	Φ_{ST} = 0.047	*

Table S4.2. Results of AMOVA for COI, and NADH2 datasets for the *T. torpedo* Sardinian sequences. *** = P-values < 0.001; ** = P-value <0.01; * = P-value <0.05. Between parentheses are the locations as shown in Figure 1. S= south, N = north, NE = northeast, W = west.

T. torpedo COI (Sardinia)			
Source of variation	Percentage of variation	Fixation Index	P-value
**One group**			
Among populations	18.86	Φ_{ST} = 0.188	*
Within populations	81.14		
**Two groups (N+W / NE+S)**			
Among groups	-45.14	Φ_{CT} = -0.451	ns
Among populations	58.72	Φ_{SC} = 0.404	ns
Within populations	86.42	Φ_{ST} = 0.136	*
**Three groups (N+NE / W/S)**			
Among groups	-197.15	Φ_{CT} = -1.971	ns
Among populations	215.65	Φ_{SC} = 0.725	ns
Within populations	81.5	Φ_{ST} = 0.185	*

T. torpedo NADH2 (Sardinia)			
**One group**			
Among populations	-3.82	Φ_{ST} = -0.038	ns
Within populations	103.82		
**Two groups (N+NW+W / NE+S)**			
Among groups	21.73	Φ_{CT} = 0.217	ns
Among populations	-21.41	Φ_{SC} = -0.273	ns
Within populations	99.69	Φ_{ST} = 0.003	ns
**Three groups (N+NE+NW / W / S)**			
Among groups	14.54	Φ_{CT} = 0.145	ns
Among populations	-9.31	Φ_{SC} = -0.109	ns
Within populations	94.77	Φ_{ST} = 0.052	ns

Table S4.3. Results of AMOVA for COI, and NADH2 datasets for the *T. nobiliana* Sardinian sequences. *** = P-values < 0.001; ** = P-value <0.01; * = P-value <0.05.

T. nobiliana COI (Sardinia)			
Source of variation	Percentage of variation	Fixation Index	P-value
**One group**			
Among populations	-90.38	Φ_{ST} = -0.904	ns
Within populations	190.38		

T. nobiliana NADH2 (Sardinia)			
**One group**			
Among populations	46.58	Φ_{ST} = 0.466	ns
Within populations	53.42		

Table S4.4 Results of AMOVA tested on the base of BAPS results for COI and NADH2 datasets for *T. marmorata*. Between parentheses are the Sardinian locations as shown in Figure 1 (S= south, SW = southwestern, N = north, NW = northwestern, E = east, NE = northeast, W = west), as well as other locations of sequences retrieved in public repositories: Atlantic Ocean (PT = Portugal, Se = Senegal) - Western Mediterranean (TY = Tyrrhenian Sea, Sp = Spain, Al = Algeria) - Central Mediterranean (Ma= Malta, SiC = Sicily, Nad =North Adriatic Sea, Sad = South Adriatic Sea) - Eastern Mediterranean (IS = Israel CY = Cyprus, Tk= Turkey, GR = Greece).

T. marmorata COI (all sequences)			
Source of variation	Percentage of variation	Fixation Index	P-value
**One group**			
Among populations	53.31	Φ_{ST} = 0.533	***
Within populations	46.69		
**Two groups (PT+Nad+SiC+N+SW+NW+W+S+E+NE+TY+Sp+Al / IS+CY+TK+GR+Sad+MA)**			
Among groups	57.58	Φ_{CT} = 0.575	***
Among populations	11.27	Φ_{SC} = 0.265	***
Within populations	31.15	Φ_{ST} = 0.688	***

T. marmorata NADH2 (all sequences)			
**One group**			
Among populations	30.95	Φ_{ST} = 0.309	***
Within populations	69.05		
**Three groups (Se / Sp+N+SW+NW+W+S+E+NE / GR+MA)**			
Among groups	55.46	Φ_{CT} = 0.555	*
Among populations	6.07	Φ_{SC} = 0.136	**
Within populations	38.47	Φ_{ST} = 0.615	***

Table S4.5. Results of AMOVA tested on the base of BAPS results for COI and NADH2 datasets for *T. torpedo*. Between parentheses are the Sardinian locations as shown in Figure 1 (S= south, N = north, NE = northeast, W = west), as well as other locations of sequences retrieved in public repositories: Atlantic Ocean (PT = Portugal, Se = Senegal) - Western Mediterranean (TY = Tyrrhenian Sea, Al = Algeria) - Central Mediterranean (SiC = Sicily, Sad = South Adriatic Sea) - Eastern Mediterranean (IS = Israel).

T. torpedo COI (all sequences)			
Source of variation	Percentage of variation	Fixation Index	P-value
**One group**			
Among populations	22.73	Φ_{ST} = 0.227	*
Within populations	77.27		
**Two groups (PT+TY+Al+N+NE+W+S / IS+Sad+SiC)**			
Among groups	12.36	Φ_{CT} = 0.123	*
Among populations	16.34	Φ_{SC} = 0.186	ns
Within populations	71.30	Φ_{ST} = 0.287	*
**Two groups (PT+TY+Al+N+NE+W+S+Sad+SiC / IS)**			
Among groups	22.78	Φ_{CT} = 0.228	ns
Among populations	13.01	Φ_{SC} = 0.168	ns
Within populations	64.21	Φ_{ST} = 0.358	*
**Three groups (PT+TY+Al+N+NE+W+S / IS / Sad+SiC)**			
Among groups	26.87	Φ_{CT} = 0.269	*
Among populations	8.43	Φ_{SC} = 0.115	ns
Within populations	64.70	Φ_{ST} = 0.353	*

T. torpedo NADH2 (all sequences)			
**One group**			
Among populations	80.23	Φ_{ST} = 0.802	ns
Within populations	19.77		
**Two groups (Se / N+NW+W+NE+S)**			
Among groups	51.02	Φ_{CT} = 0.510	ns
Among populations	2.79	Φ_{SC} = 0.057	ns
Within populations	46.20	Φ_{ST} = 0.538	ns

Table S4.6. Results of AMOVA tested on the base of BAPS results for COI and NADH2 datasets for *T. nobiliana*. Between parentheses are the Sardinian locations as shown in Figure 1 (S= south, E = east, W = west), as well as other locations of sequences retrieved in public repositories: Atlantic Ocean (CA = Canada, PT = Portugal, USA) - Western Mediterranean (TY = Tyrrhenian Sea) - Central Mediterranean (SiC = Sicily) - Saf = South Africa - AUS = Australia, NZ = New Zealand, Tas = Tasman Sea - Na = not available.

T. nobiliana COI (all sequences)			
Source of variation	Percentage of variation	Fixation Index	P-value
**One group**			
Among populations	63.36	Φ_{ST} = 0.633	***
Within populations	36.64		
**Two groups (Na+Saf+AUS+NZ+PT+SiC+TY+E+S+W / CA)**			
Among groups	91.53	Φ_{CT} = 0.915	ns
Among populations	3.05	Φ_{SC} = 0.359	**
Within populations	5.42	Φ_{ST} = 0.946	***

T. nobiliana NADH2 (all sequences)			
**One group**			
Among populations	83.95	Φ_{ST} = 0.839	***
Within populations	16.05		
**Two groups (Na+Tas+Saf+E+S+W / USA)**			
Among groups	79.60	Φ_{CT} = 0.796	ns
Among populations	14.66	Φ_{SC} = 0.718	**
Within populations	5.75	Φ_{ST} = 0.942	***
